# Cognitive Correlates of Resilience in Adults Experiencing Homelessness

**DOI:** 10.1093/arclin/acaf018

**Published:** 2025-03-04

**Authors:** Caitlin M Terao, Michelle J Blumberg, Suzanne Mckeag, Vicky Stergiopoulos, Stephen W Hwang, Kristina M Gicas

**Affiliations:** Department of Psychology, York University, Toronto, Canada; Department of Psychology, York University, Toronto, Canada; Department of Psychology, York University, Toronto, Canada; General Adult Psychiatry and Health Systems Division, Centre for Addiction and Mental Health, Toronto, Canada; Department of Psychiatry, University of Toronto, Toronto, Canada; MAP Centre for Urban Health Solutions, Li Ka Shing Knowledge Institute St. Michael’s Hospital, Unity Health Toronto, Toronto, Canada; Division of General Internal Medicine, Department of Medicine, University of Toronto, Toronto, Canada; Department of Psychology, York University, Toronto, Canada; Department of Psychology, University of the Fraser Valley, Abbotsford, Canada

**Keywords:** Assessment, Cross-cultural/minority, Executive functions, Intelligence

## Abstract

**Objective:**

In adults who have experienced homelessness, greater psychological resilience is related to better quality of life, community functioning, and social cognition. Domain-specific cognitive functioning is positively associated with resilience in housed populations; however, these relationships have yet to be explored among adults experiencing homelessness. The aim of this study is to examine the relationships between domain-specific cognitive function and psychological resilience among adults experiencing homelessness.

**Method:**

One hundred and six adults who have experienced homelessness were recruited in Toronto, Canada, and 88 were included in analyses (51% female, mean age = 43 years). Study measures assessed psychological resilience as well as domain-specific cognition (vocabulary, oral reading, processing speed, episodic memory, and executive functioning) using the NIH Toolbox Cognition Battery. Additional covariates of interest included psychological distress, social network size, substance misuse, and major psychiatric disorders. Hierarchical regression modeling explored the contributions of each cognitive domain to resilience while accounting for established covariates.

**Results:**

Oral reading was positively associated with higher resilience, explaining 12.45% of the variance in resilience while controlling for age, education, gender, substance misuse, psychological distress, and social network size. Performance on measures of executive functioning, processing speed, and visual memory were not found to be related to self-reported resilience.

**Conclusion:**

The results suggest that verbal vocabulary, shaped by the accumulation of experiences across one’s lifetime, may be an important contributor to psychological resilience. Better crystallized abilities may reflect more enriched early life experiences that are critical to better coping skills and well-being of adults experiencing homelessness.

## INTRODUCTION

Homeless and precariously housed adults are systematically disadvantaged and overexposed to trauma, which has serious negative implications well beyond the experience of being unhoused. The experience of homelessness itself has been shown to be a traumatic event with enduring mental health repercussions (Tsai et al., 2020). Individuals experiencing homelessness are at an increased risk of multimorbid mental and physical illness (i.e., the presence of two or more chronic conditions) as well as premature mortality (Aldridge et al., 2018; Roncarati et al., 2018; Vila-Rodriguez et al., 2013). Furthermore, homelessness is associated with chronic stress, social exclusion, and healthcare inequity, all of which can negatively impact cognitive functioning (Fazel et al., 2014; Luchenski et al., 2018; Mullins et al., 2021). Past research has predominantly focused on risk factors for poor outcomes among homeless adults, highlighting the need for more research on positive coping factors that are protective against the extreme adversity associated with being unhoused. To better understand the factors that contribute to strengthening resilience, we examined the relationships between self-reported psychological resilience and select cognitive domains in adults who have experienced homelessness.

### Cognitive Functioning and Homelessness

Cognitive functioning can be broadly divided into fluid and crystalized cognitive abilities, with fluid cognition referring to the ability to effectively interact and problem-solve with novel information, whereas crystallized cognition refers to intellectual abilities that utilize prior knowledge derived from accumulated life experience (Cattell, 1963). Approximately 25% of homeless adults experience global cognitive impairment (Depp et al., 2015), with up to 82% of this population exhibiting impaired fluid cognitive abilities (Burra et al., 2009). Previous research has documented various domain-specific impairments in inhibition, attention, decision-making, processing speed, executive functioning, and learning and memory among adults experiencing homelessness using a wide variety of traditional neuropsychological measures (Ennis et al., 2015; Gicas et al., 2020; Stergiopoulos et al., 2015; Stone et al., 2019). Impaired cognitive functioning is particularly relevant within this population, as it has been shown to be related to decreased functional capacity and poorer everyday outcomes (Gicas et al., 2021; Mahmood et al., 2021; Maye et al., 2023). Taken together, elevated rates of domain-specific cognitive difficulties are observed in adults experiencing homelessness and this has important implications for this population’s daily functioning, although cognitive impairment is not the focus of the present study. Combined with the multifaceted social and individual-level inequities associated with the experience of homelessness highlighted above, cognitive abilities may uniquely impact aspects of psychological functioning, such as resilience, in adults experiencing homelessness or precarious housing.

### Psychological Resilience and Homelessness

Psychological resilience is typically conceptualized as adversity followed by a positive adaptation or outcome (Hiebel et al., 2021). Resilience has been inconsistently defined as a trait-level characteristic, a positive outcome variable, and a dynamic process; however, current research typically describes resilience as an ongoing multidimensional process (Métais et al., 2022; Vella & Pai, 2019). Higher resilience has been shown to relate to greater social network size and lower psychological distress (Bacchi & Licinio, 2017; Barahmand & Ahmad, 2016; Park et al., 2021; Riehm et al., 2021). The capacity to sustain a higher level of resilience may be particularly critical for adults experiencing homelessness given the traumatic and stressful experiences this population is exposed to on an ongoing basis (Deck & Platt, 2015; Ellsworth, 2019). Adults experiencing homelessness, despite disproportionate trauma exposure, have been shown to demonstrate typical patterns of positive adaptation following trauma (Stump & Smith, 2008). Using a qualitative approach, Phipps et al. (2021) found that resilience helps women overcome the trauma associated with homelessness. Further, higher levels of resilience are associated with better self-reported quality of life in homeless adults (Mejia-Lancheros et al., 2021). Adults experiencing homelessness with higher self-reported levels of resilience have also been shown to have superior community functioning, greater social support, more days stably housed, and reduced symptoms of psychological distress (Durbin et al., 2019; Greenberg et al., 2019). Greater resilience has also been shown to be related to reduced suicidal ideation among homeless youth (Cleverley & Kidd, 2011). It is important to understand the cognitive correlates of resilience in homeless adults given the elevated levels of cognitive impairment and the relevance of resilience to the well-being of this population. Understanding the cognitive contributions to resilience expands opportunities to foster resilience within this marginalized population through these modifiable targets.

### Cognitive Functioning and Resilience

The cognitive appraisal of resilience (CAR) model has been proposed as a potential theoretical basis for the role of cognitive functioning in resilience (Yao & Hsieh, 2019). Under this model, resilience is defined through the core concepts of adverse experiences, cognitive appraisal, and positive adaptation. Cognitive appraisal, comprised of pain perception cognitive-emotional processes, is a critical moderator within this model and is proposed to mitigate the negative impacts of adverse events on positive adaptation. This theory posits that cognitive flexibility, a dimension of executive functioning, moderates the relationship between resilience and adverse events by shifting attention between the two aspects of cognitive appraisal, pain perception and cognitive-emotional processes (Yao & Hsieh, 2019). Consistent with this model, a systematic review concluded that cognitive flexibility plays a critical role in resilience under most definitions of the construct (Hiebel et al., 2021).

Domain-specific cognitive functioning has been proposed to support resilience within clinical and non-clinical and clinical populations, such as individuals with mental and physical health disorders (Métais et al., 2022). Consistent with the CAR model, greater executive functioning has been shown to be related to higher resilience across the adult lifespan in healthy individuals and individuals with HIV, sleep disorders, post-traumatic stress disorder, schizophrenia, bipolar disorder, and depression (Deng et al., 2018; Fazeli et al., 2019; Han et al., 2021; Laird et al., 2019; Simeon et al., 2007). Within the domain of executive functioning, cognitive flexibility specifically has been shown to predict resilience in healthy adults (Genet & Siemer, 2011). Faster processing speed has also been found to relate to greater resilience in healthy individuals and individuals with HIV, sleep disorders, post-traumatic stress disorder, schizophrenia, bipolar disorder, and depression (Deng et al., 2018; Fazeli et al., 2019; Han et al., 2021; Laird et al., 2019; Simeon et al., 2007). Prior work has shown that verbal and visual memory relate to higher resilience (Fazeli et al., 2019; Han et al., 2021). Visual memory was also shown to positively relate to resilience in a community-based study involving highly traumatized adults (Wingo et al., 2010). Several fluid cognitive domains appear to relate to resilience positively; however, the nature of this relationship among adults experiencing homelessness remains understudied.

Few studies have explored the relationship between resilience and cognition in homeless adults. Gicas et al. (2021) found no evidence that resilience moderated the relationship between cognition and functional outcomes in homeless adults with serious mental illness; however, the relationship between resilience and cognition was not directly examined. Greenberg et al. (2019) did not find evidence of a relationship between resilience and cognition in male veterans experiencing homelessness; however, this study used a global composite score for cognition. Given the domain-specific patterns of cognitive impairment associated with the experience of homelessness (Stergiopoulos et al., 2015), it is important to examine how specific cognitive domains relate to resilience in adults experiencing homelessness. Additionally, associations between resilience and cognitive functioning have yet to be explored in adults experiencing homelessness who are non-veterans and who identify as female.

### Current Study

The current study aimed to gain a deeper understanding of the relationship between resilience and variations in domain-specific cognitive functioning among adults experiencing homelessness and housing instability in a large Canadian urban center. We aimed to recruit roughly even numbers of men and women as well as an equal age representation across the adult lifespan (i.e., younger, middle aged, and older adults) to increase generalizability of study findings and better capture the diverse lived experiences among adults experiencing homelessness and housing instability. We hypothesized that greater performance on measures of fluid cognition will be related to higher psychological resilience while performance on measures of crystallized cognition will not be significantly related to resilience in adults who experience homelessness. Specifically, we hypothesized that greater performance on measures of executive functioning, processing speed, and visual memory will be related to higher self-reported resilience above and beyond known important correlates of resilience. In addition to key demographic factors, established correlates of resilience include social network size and psychological distress (Cronley & Evans, 2017; Durbin et al., 2019; Liu et al., 2020). We additionally predicted that cognitive flexibility would have the strongest relationship with psychological resilience among the examined cognitive domains. A more nuanced understanding of the relationship between specific cognitive domains and resilience will inform targeted interventions to improve coping capacity and quality of life in adults experiencing homelessness.

## METHODS

### Participants

**Fig. 1:**
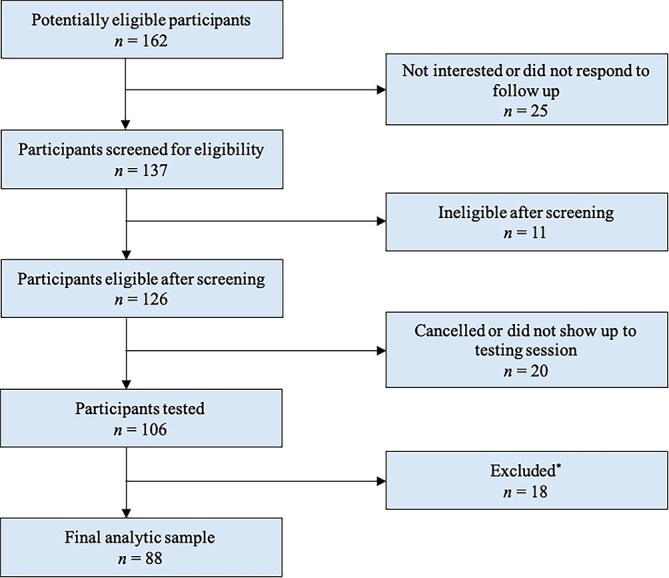
Flow chart of recruitment process. ^*^Participants were excluded from the analytic sample for failing to complete study measures (*n* = 9), unclear or unknown educational attainment (*n* = 3; e.g., “Can’t remember”), declining to provide their age (*n* = 1), and based on experimenter-rated invalid performance for any measure included in the regression models (*n* = 5).

A sample of adults experiencing homelessness or precarious housing (*n* = 106, 46% female, ages 19–70 years) was recruited between September 2022 and July 2023 from a list of individuals who consented to be re-contacted for future research after participating in the Ku-gaa-gii pimitizi-win community-based cohort study (Richard et al., 2022), through word of mouth, and from shelters in the Toronto area, including a drop-in center for older men, a women’s only residence, and a temporary COVID-19 shelter site. Inclusion criteria were as follows: (1) being at least 18 years old; (2) self-reported English fluency; (3) the ability to see and hear testing material; (4) having experienced at least two episodes (at least seven consecutive nights) of homelessness or precarious housing in the past year. Participants who self-reported gross cognitive impairment, demonstrated evidence during the consent process that they were unable to understand the nature of the testing session, or who were unable or unwilling to appropriately follow testing instructions were excluded. See Fig. 1 for a flow chart of the recruitment process. All participants provided written informed consent and received modest financial compensation for their involvement. Ethics approval for the current study and the honorarium amount for participation were obtained from the Research Ethics Boards at York University and St. Michael’s Hospital. The Ethics Boards take into account: the local socioeconomic conditions at the time of the study, the economic situation of the participants likely to be recruited, the goal of protecting the autonomy of the participants (i.e., require providing cash honoraria rather than gift cards), and balance the amount of time required for assessments with the size of the honorarium provided.

Participants were excluded from the analytic sample for failing to complete study measures (*n* = 9), unclear or unknown educational attainment (*n* = 3; e.g., “Can’t remember”), declining to provide their age (*n* = 1), and based on experimenter-rated invalid performance for any measure included in the regression models (*n* = 5). Testing session notes were reviewed for these five cases and the participants were excluded due to: self-reported random responding (*n* = 1), falling asleep during the testing session (*n* = 1), alcohol consumption during test administration (*n* = 2), and being unable to read the Oral Reading Recognition Test stimuli (*n* = 1). The final analytic sample included 88 individuals experiencing homelessness or precarious housing.

### Procedures

Participant testing was conducted in private spaces at either York University, a local inner-city library, or the shelters where recruitment took place. After providing informed consent, participants completed questionnaires on demographics, resilience, psychological distress, and social network size through the survey platform REDCap (https://www.project-redcap.org/) delivered on an iPad. Trained research assistants, supervised by a clinical psychologist, then administered a brief assessment of major mental disorders. Participants subsequently completed objective measures of cognitive functioning before being given the chance to ask questions. The testing session typically lasted two hours with a brief break approximately halfway through the assessment. In terms of the approximate duration of the measures, the questionnaires required 30 min, brief assessment of major mental disorders required 40 min, and objective cognitive measures required 40 min with roughly 15 min for consent and questions. Participants were provided additional breaks as needed throughout the testing session (e.g., due to fatigue).

Immediately upon completion of testing, the test administrator subjectively rated whether there were any extraneous factors that impacted the integrity of the questionnaire and neurocognitive data using a 5-point Likert scale ranging from “Clearly Invalid” to “Clearly Valid”. Invalid ratings were primarily due to failure to comply with testing instructions, extreme fatigue, intoxication, suboptimal effort, comprehension difficulties, and early voluntary termination of the testing session. Data rated as “Likely Valid” or higher were retained for analyses. For data rated “Questionably Valid”, notes of the testing session were reviewed, and the inclusion of data was decided on a case-by-case basis after discussion by the research team. This qualitative approach has been taken in several other studies conducted in samples of adults experiencing homelessness to ensure that the data is of a sufficient quality to include in statistical analyses (e.g., Blumberg et al., 2024; Gicas et al., 2017, 2020).

### Materials

#### Psychological resilience

Self-reported psychological resilience was assessed using the abbreviated Connor-Davidson Resilience Scale (CD-RISC2), which is a two-item (“Able to adapt when changes occur” and “Tend to bounce back after illness, injury, or other hardship”) measure of resilience that uses a five-point Likert style scale (“Not true at all”, “Rarely true”, “Sometimes true”, “Often true”, and “True nearly all the time”) (Vaishnavi et al., 2007). Possible scores range from zero to eight, with higher scores indicating greater psychological resilience. The CD-RISC2 is significantly correlated with the full scale and has demonstrated good internal consistency, test–retest reliability, and convergent and discriminant validity (Connor & Davidson, 2003; Vaishnavi et al., 2007). This measure has been used as a main outcome measure of resilience across diverse areas of research and has been administered to healthy adults, adults experiencing homelessness, and individuals with cancer, depression, generalized anxiety disorder, and posttraumatic stress disorder (Huffman et al., 2021; Ludolph et al., 2019; Mejia-Lancheros et al., 2021; Vaishnavi et al., 2007; West et al., 2020).

#### Cognition

Cognition was assessed using the NIH Toolbox Cognitive Battery, for which the measures, scoring algorithms, and norms are publicly available (https://nihtoolbox.org/). The NIH Toolbox Cognitive Battery includes seven subtests measuring fluid and crystallized cognition (Hodes et al., 2013). The battery has five subtests of fluid abilities. The Flanker Inhibitory Control and Attention Test is a measure of executive functioning, specifically attention and inhibitory control. The Dimensional Change Card Sort Test measures cognitive flexibility, another dimension of executive functioning. Both tests are scored based on accuracy and reaction time; specifically, if accuracy is $\le$80%, the final score will be equal to the accuracy score, whereas reaction time and accuracy scores are combined for accuracy levels >80%. The List Sorting Working Memory Test measures working memory, an aspect of executive functioning, and is scored based on total correctly recalled items. The Pattern Comparison Processing Speed Test assesses processing speed using the number of correct answers within 85s. The Picture Sequence Memory Test measures visual episodic memory. Scores are automatically derived by converting the number of correct adjacent pairs across trials one and two to a theta score.

The remaining two subtests measure crystallized abilities. The Oral Reading Recognition Test is a measure of reading ability, and the Picture Vocabulary Test is a measure of general vocabulary. Both tests are scored using item response theory theta scores (Hodes et al., 2013). There are also two demographically adjusted composite scores available for Fluid Cognition and Crystallized Cognition; however, these are not the focus of the present study.

Each of the seven subtests of the NIH Toolbox Cognitive Battery have been shown to have strong psychometric properties among cognitively healthy adults, including adequate to strong evidence for test–retest reliability, convergent validity, discriminant validity, and construct validity (Carlozzi et al., 2014; Dikmen et al., 2014; Gershon et al., 2014; Tulsky et al., 2014; Zelazo et al., 2014). The NIH Toolbox Cognitive Battery has also been shown to be valid to use across a broad range of clinical populations, including individuals with traumatic brain injury, substance use disorder, and multimorbid mental illness (Carlozzi et al., 2017; Holdnack et al., 2017; Jett et al., 2023; Tulsky et al., 2017).

#### Psychological distress

Psychological distress was measured using the Hospital Anxiety and Depression Scale (HADS), which is a self-report measure of anxiety and depressive symptomatology (Zigmond & Snaith, 1983). A meta-confirmatory factor analysis concluded that the anxiety and depression subscales of the HADS should be combined into a single index of psychological distress (Norton et al., 2013). When the two subscales are combined, possible scores range from zero to 42, with a score greater than 22 indicating potentially clinically significant psychological distress. HADS has been used extensively in community-based and clinical samples as a measure of psychological distress, demonstrating adequate to strong test–retest reliability, internal consistency, structural validity, construct validity, convergent validity, and discriminant validity (Bjelland et al., 2002; Norton et al., 2013; Stern, 2014).

#### Social network size

Social network size was measured using the Lubben Social Network Scale 6-item (LSNS-6), which is a self-report measure of social engagement with friends and family members (Lubben & Gironda, 2000). Possible scores range from zero to 30, with higher scores indicating greater social engagement. LSNS-6 has been shown to have good internal consistency, high person and item reliability, and a positive relationship with physical and cognitive health indicating good convergent validity (Gray et al., 2016; Merchant et al., 2020).

#### Mental health disorders

Trained research assistants administered the Mini-International Neuropsychiatric Interview (M.I.N.I.), which is a brief diagnostic interview designed to assess current and past major psychiatric disorders (Sheehan et al., 1998). The M.I.N.I. has been shown to have excellent inter-rater reliability, very good test–retest reliability, and good to strong criterion validity in individuals with and without mental health disorders (Sheehan et al., 1998). The present study examined current diagnoses of major depressive disorder, generalized anxiety disorder, psychotic disorders, bipolar disorder, antisocial personality disorder, posttraumatic stress disorder, obsessive-compulsive disorder, social anxiety disorder, panic disorder, agoraphobia, substance use disorder, and alcohol use disorder. Substance use disorder and alcohol use disorder were additionally classified as mild, moderate, or severe based on the number of symptoms endorsed for each disorder.

#### Demographics

Participants completed a demographic questionnaire indicating their age, gender identity, ethnicity, native language, and years of formal education. Participants self-reported their gender identity by selecting any of the following options: male, female, intersex, trans-female to male, trans-male to female, prefer not to answer, do not know, and other. Participants self-reported their ethnicity by selecting as many of the following options as applied: Asian–East, South, Southeast; Black–African, Caribbean, North American; White–European, North American; Indian–Caribbean; Latin American; Middle Eastern; First Nations; Inuit; Métis; Indigenous/Aboriginal not included elsewhere; mixed heritage; other; prefer not to answer; and do not know.

### Statistical Analyses

Descriptive statistics were used to characterize performance on all measures in the full sample (Tables 1 and 2). For descriptive purposes, Spearman’s correlations examined the zero-order correlations between resilience and model covariates and each NIH cognitive test score due to non-normal distributions (Table 2). A series of hierarchical regression models explored the contributions of each cognitive subtest to resilience while accounting for known covariates of resilience (Bacchi & Licinio, 2017; Barahmand & Ahmad, 2016; Park et al., 2021; Riehm et al., 2021). The base regression model included gender (female:male), education (years), age (years), substance misuse (met criteria:did not meet criteria), psychological distress (raw scores), and social network size (raw scores) as predictors of resilience. Gender was included as a dichotomous categorical variable as only the female and male categories were endorsed by participants. Substance misuse was included as a dichotomous categorical variable based on whether participants did or did not meet M.I.N.I. criteria for alcohol and/or substance use disorder. Neuropsychological test performance was characterized using age-corrected standardized scores for descriptive purposes and raw scores were used for all other analyses. The full regression model included all base predictors (i.e., covariates) with the addition of a single cognitive score, for a total of seven separate full models predicting resilience. ANOVAs were used to compare each full model to the base model. Model assumptions were evaluated, including investigating the normality of residuals, variance inflation, multicollinearity, and multivariate normality. As sensitivity analyses, all models were re-run without outliers (*M*  $\pm$ 3 *SD*) and re-run with age-corrected standardized scores for each NIH cognitive subtest score. Given there were no notable differences in results between any of the models, the original model results are reported here. As an additional sensitivity analysis, all analyses were run including participants who were excluded based on subjectively rated invalid performances (*n* = 5)*.* Results did not change with the inclusion of these individuals, so the cleaned sample is reported here. Benjamini–Hochberg False Discovery Rate Method was used to control the false discovery rate across the seven nested model comparisons (Benjamini & Hochberg, 1995). Squared partial correlations were used to quantify the effect size for statistically significant cognitive predictors of resilience. As a follow-up analysis to ensure the robustness of study findings, all seven NIH cognitive subtest scores were entered into a regression model with base predictors (i.e., covariates) with resilience as the outcome variable. Data were analyzed using R version 4.3.1 (R Core Team, 2020).

**Table 1:** Categorical sample characteristics (*N* = 88)

Variable	Overall sample (*N* = 88)	
Categorical variables	*n*	%
Gender^a^		
Female	45	51.1
Male	43	48.9
Ethnicity/Race		
White North American	28	31.8
Black African	23	26.1
White European	13	14.8
South Asian	8	9.1
Black Caribbean	7	8.0
Mixed heritage	6	6.8
Other	3	3.4
Housing status		
Emergency shelter	50	56.8
Transitional housing program	12	13.6
Own apartment or house	11	12.5
Hotel or motel	7	8.0
Family member or friend’s residence	4	4.6
Street or outdoor location	3	3.4
Group home	1	1.1
Current mental health disorders		
Substance use disorder	32	36.4
Alcohol use disorder	23	26.1
Antisocial personality disorder	18	20.5
Psychotic disorders^b^	17	19.3
Major depressive disorder	16	18.2
Posttraumatic stress disorder	14	15.9
Obsessive-compulsive disorder	14	15.9
Social anxiety	12	13.6
Panic disorder	12	13.6
Agoraphobia	10	11.4
Generalized anxiety disorder	6	6.8
Bipolar disorder	6	6.8
English second language	21	23.9

^a^No other gender categories were endorsed.

^b^Includes major depressive disorder with psychotic features (*n* = 4).

**Table 2:** Continuous sample characteristics and correlations with resilience (*N* = 88)

Continuous variables	M (SD)	Range	Correlation^a^
Age	43.1 (14.2)	19–70	−0.11
Years of education	13.6 (2.5)	8–21	0.04
Resilience (CD-RISC2)	5.7 (1.8)	1–8	–
Social Network (LSNS-6)	9.8 (6.3)	0–30	.25^*^
Psychological Distress (HADS)	14.9 (7.4)	1–37	−.53^***^
Dimensional change	83.5 (15.3)	54–141	.23^*^
Flanker	73.1 (11.2)	54–103	0.13
List sorting	90.6 (14.2)	61–126	0.15
Pattern comparison	86.1 (21.9)	54–135	.22^*^
Picture sequence	92.3 (14.1)	69–142	0.15
Picture vocabulary	87.7 (14.1)	59–118	0.06
Oral reading	102.8 (19.4)	54–146	.23^*^

*Note. M* = mean; *SD* = standard deviation; CD-RISC2 = abbreviated Connor-Davidson Resilience Scale; LSNS-6 = Lubben Social Network Scale 6-item; HADS *=* Hospital Anxiety and Depression Scale.

The *M (SD)* and *Range* presented for the NIH Cognitive Subtest Scores are age-corrected standardized scores. The correlations presented are for uncorrected scores and CD-RISC2.

^a^Spearman’s zero-order correlations (*df* = 86) with Resilience (CD-RISC2)

^*^ *p* < .05. ^**^p < .01. ^***^p < .001.

## RESULTS

The sample demographics and clinical characteristics are presented in Tables 1 and 2. The final analytic sample included 88 adults experiencing homelessness with an average age of 43.05 years (*SD* = 14.22, range = 19–70) and an approximately even gender division. Seventy-four percent of the final sample met criteria for at least one mental health disorder, with the most prevalent being substance use disorder, alcohol use disorder, antisocial personality disorder, psychotic disorders, and major depressive disorder. Among those who met criteria for substance use disorder, 9% were classified as mild, 22% as moderate, and 70% as severe. Among those who met criteria for alcohol use disorder, 35% were classified as mild, 22% as moderate, and 43% as severe. An additional 33% of participants were currently experiencing suicidal thoughts, actions, or intentions. Given the high rate of multimorbid mental illness observed in the present sample, Welch’s *t*-tests were used to investigate differences in performance on the NIH cognitive subtests among participants who met and did not meet criteria for at least one mental health disorder. Results indicated no significant differences in performance across the seven NIH cognitive subtests (all *p* > .05), so this variable (met criteria:did not meet criteria) was not included as a covariate in the base regression model.

**Fig. 2:**
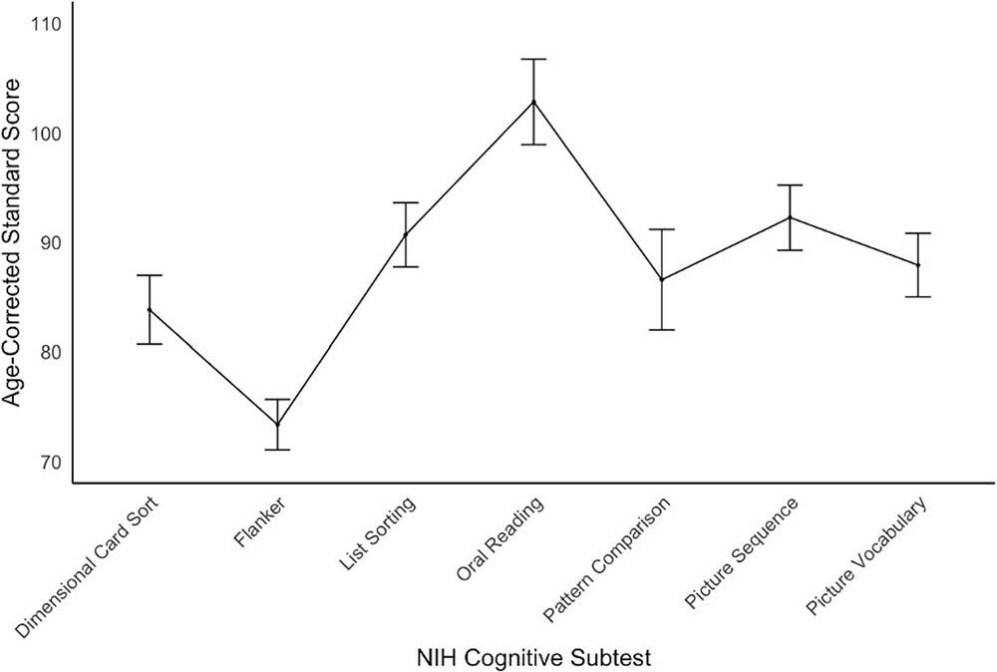
Mean age-corrected standardized cognitive scores with 95% confidence intervals.

Overall, the sample performed within the Below Average to Average range relative to age-matched norms on the NIH cognitive subtests (Guilmette et al., 2020). See Table 2 for descriptive statistics of age-corrected standardized NIH cognitive subtest scores. Participants performed in the Average range on the List Sorting Working Memory Test, Picture Sequence Memory Test, and Oral Reading Recognition Test. Participants performed in the Low Average range on the Dimensional Change Card Sort Test, Pattern Comparison Processing Speed Test, and Picture Vocabulary Test. Participants were Below Average on the Flanker Inhibitory Control and Attention Test. See Fig. 2 for a graphical depiction of age-corrected standardized NIH cognitive subtest scores.

For descriptive purposes, rank order correlations were calculated between resilience and all regression model variables (Table 2). In terms of base regression model covariates, correlational analyses indicated higher CD-RISC2 scores were significantly related to greater LSNS-6 scores and lower HADS scores. CD-RISC2 scores were not found to be significantly related to gender, age, education, or substance misuse. In terms of the NIH cognitive subtest scores, correlation analyses indicated higher CD-RISC2 scores were significantly related to greater performance on the Oral Reading Recognition Test, Dimensional Change Card Sort Test, and Pattern Comparison Processing Speed Test. CD-RISC2 scores were not found to be significantly related to performance on the Flanker Inhibitory Control and Attention Test, List Sorting Working Memory Test, Picture Sequence Memory Test, or Picture Vocabulary Test.

In the base regression model, gender, age, education, substance misuse, LSNS-6, and HADS were entered as independent variables, with CD-RISC2 as the outcome variable. Results indicated that 28% of the variability in CD-RISC2 scores can be explained by this model (R^2^ = 0.282, *p* < .001). The HADS score was the only significant predictor of resilience within the model whereby higher psychological distress was associated with lower resilience (*B* = −0.13, *p* < .001). See Table 3 for the full base model results.

For the full regression models, each NIH cognitive subtest score was entered into a separate multiple regression model predicting CD-RISC2 along with gender, age, education, substance misuse, LSNS-6, and HADS, which was then compared to the base model. Contrary to our hypothesis, the Dimensional Change Card Sort Test, Flanker Inhibitory Control and Attention Test, List Sorting Working Memory Test, Pattern Comparison Processing Speed Test, and Picture Sequence Memory Test were not significantly (*p* > .05) associated with CD-RISC2 in their respective regression models. See Table 3 for the cognitive coefficients for each of the seven regression models and the supplemental material for the full model results.

The Picture Vocabulary Test (*B* = 0.18, *p* = .041) was a significant predictor of CD-RISC2 when entered into a multiple regression model along with gender, age, education, substance misuse, LSNS-6, and HADS (R^2^ = 0.319, *p* < .001). Nested model comparisons showed that the addition of the Picture Vocabulary Test was significantly related to resilience beyond the base model covariates, *F*(1,80) = 4.33, *p* = .041; however, this was no longer significant after Benjamini–Hochberg False Discovery Rate Method was used, *p_BH_* = 0.142.

The Oral Reading Recognition Test (*B* = 0.18, *p* = .001) was a significant predictor of CD-RISC2 within its respective multiple regression model (R^2^ = 0.37, *p* < .001), which was in contrast to our initial hypothesis. Nested model comparisons showed that the Oral Reading Recognition Test was significantly related to resilience beyond the base model covariates, *F*(1, 80) = 11.23, *p* = .001, *p_BH_* = 0.009. Squared partial correlation showed that the Oral Reading Recognition Test explained 12.45% of the variance in CD-RISC2 while controlling for the influence of model covariates.

To further reduce the risk of type 1 error and ensure the robustness of study findings, we included all cognitive subtests and covariates within a single regression model predicting resilience. The Oral Reading Recognition Test (*B* = 0.16, *p* = .018) was the only significant cognitive predictor within this model. See the supplemental material for the full model results. Due to issues with multicollinearity, the separate models are reported here.

Based on the observed pattern of results, additional supplementary analyses were conducted in which Crystallized Composite, Fluid Composite, and Crystallized-Fluid Difference (i.e., Crystallized minus Fluid, representing decline from premorbid levels of cognitive functioning; Iverson et al., 2023) scores were entered into separate multiple regression models predicting CD-RISC2 along with gender, age, education, substance misuse, LSNS-6, and HADS. In line with the above findings, the Crystallized Composite score (*B* = 0.06, *p* = .002) was a significant predictor of CD-RISC2 within its respective multiple regression model (R^2^ = 0.36, *p* < .001), whereas the Fluid Composite and Crystallized-Fluid Difference scores were not significantly (*p* > .05) associated with CD-RISC2 scores. See the supplemental material for full model results.

**Table 3:** Hierarchical regression results with resilience as the outcome (*N* = 88)

Predictors	Estimate	*SE*	95% CI		*p* (*p_BH_*)
			LL	UL	
Step 1: Base model					
Female gender	0.29	0.38	−0.47	1.05	0.448
Age	<0.01	0.01	−0.02	0.03	0.863
Education	<0.01	0.07	−0.14	0.14	0.975
Substance misuse	0.37	0.39	−0.41	1.15	0.345
**HADS**	**−0.13**	**0.03**	**−0.18**	**−0.07**	**<0.001**
LSNS-6	0.01	0.03	−0.05	0.07	0.696
Step 2: cognitive coefficients^a^					
Dimensional change	0.06	0.14	−0.21	0.34	0.642 (0.642)
Flanker	0.13	0.18	−0.24	0.49	0.494 (0.642)
List sorting	0.09	0.05	−0.02	0.21	0.112 (0.196)
Pattern comparison	0.02	0.01	<−0.01	0.04	0.094 (0.196)
Picture sequence	0.14	0.21	−0.28	0.55	0.514 (0.642)
Picture vocabulary	0.18	0.09	0.01	0.35	0.041 (0.142)
**Oral Reading** ^b^	**0.18**	**0.05**	**0.07**	**0.29**	**0.001 (0.009)**

*Note.* CI = confidence interval; *LL* = lower limit; *UL* = upper limit; HADS = Hospital Anxiety and Depression Scale total score; LSNS-6 = Lubben Social Network Scale 6-item. Bold indicates statistical significance (*p*<.05).

^a^Each cognitive score was entered into a separate regression model with the base model covariates. Only the cognitive score for each of the seven models are reported here. See the supplemental material for full model results.

^b^Oral Reading Recognition Test, *r^2^_p_* = 0.1245.

## DISCUSSION

The present study was the first to examine the relationship between domain-specific cognition and psychological resilience among adults experiencing homelessness and housing instability. Surprisingly, performance on measures of executive functioning, processing speed, and visual memory, which were predicted to positively relate to resilience, were not significantly associated with self-reported resilience. Also contrary to study hypotheses, better oral reading, an aspect of crystallized cognition, was significantly associated with higher psychological resilience over and above the effects of age, education, gender, substance misuse, psychological distress, and social network size, explaining 12.45% of the variance in resilience while controlling for model covariates. Greater picture vocabulary scores, the other crystallized measure, were also associated with higher psychological resilience, although this relationship was no longer significant after controlling for multiple comparisons. In terms of the simple relationships between resilience and key study variables, higher resilience was found to be significantly associated with lower psychological distress and greater social network size, which is consistent with past research (Cronley & Evans, 2017; Durbin et al., 2019; Liu et al., 2020). Higher resilience also related to greater performance on measures of processing speed and cognitive flexibility; however, these simple relationships were not significant after the addition of important covariates. The current study identified oral reading abilities as a novel correlate of resilience among adults experiencing homelessness and housing instability while finding no evidence of a relationship between any domain of fluid cognition and resilience, the implications of which are discussed below.

In contrast to past research among housed populations, performance on measures of executive functioning, processing speed, or visual memory were not found to be significantly related to psychological resilience beyond important correlates within the current study (Deng et al., 2018; Fazeli et al., 2019: Laird et al., 2019; Han et al., 2021). Also inconsistent with past research, cognitive flexibility, which was predicted to show the strongest relationship with resilience, similarly demonstrated no evidence of a relationship beyond established covariates (Genet & Siemer, 2011; Hiebel et al., 2021). These discrepancies are likely not attributable to a restricted range of cognitive performance, as scores on each NIH cognitive subtest ranged from severe impairment to very superior performance, which suggests sufficient performance variability to capture these established relationships. Instead, these inconsistencies suggest that the relationship between resilience and domain-specific cognition may differ among adults who have experienced homelessness compared to previous work with stably housed populations. Indeed, Greenberg et al. (2019) similarly found no evidence of a relationship between resilience and a global cognitive composite score based on measures of attention, processing speed, problem solving, working memory, verbal memory, and visual memory in a sample of male homeless veterans. In the context of the CAR model of resilience, cognitive control is specified as having a modulatory effect on cognitive flexibility, whereby impairments in top-down cognitive control processes may negatively impact cognitive flexibility (Yao & Hsieh, 2019). Thus, in the context of the current study findings, it is possible that the observed cognitive control impairment in this sample (Below Average, 4th percentile) was of sufficient magnitude that it impaired efficiency in cognitive flexibility and undermined the modulatory role that cognitive appraisal ultimately has on psychological resilience. Instead, adults experiencing homelessness may need to compensate by relying on alternative cognitive systems to support resilience. Performance was greatest for the oral reading measure compared to performance on the other neurocognitive measures, suggesting participants may have relied on their strongest cognitive abilities to foster resilience.

The present study identified oral reading abilities, an aspect of crystallized cognition, as a novel correlate of resilience within adults experiencing homelessness. Contrary to study findings, Greenberg et al. (2019) found no evidence of a relationship between resilience and a premorbid verbal intelligence composite among homeless male veterans, which was measured through mathematics, comprehension, reading ability, and vocabulary. Moreover, there is limited evidence in the literature to suggest a clear relationship between measures of premorbid intelligence and resilience in housed populations, with most studies finding no evidence of a relationship (Conder et al., 2015; Friborg et al., 2006; Hall & Theron, 2016). It is possible this discrepancy may reflect differences in construct measurement and sample characteristics between studies. In the context of our findings, better crystallized cognitive abilities may be considered a proxy for more enriched earlier experiences, which have been shown to contribute to enhanced coping abilities later in life (Daskalakis et al., 2013; Langenhof & Komdeur, 2018) and, therefore, may help to explain the observed relationship between resilience and crystallized cognition. Further research is needed to explore associations between resilience, crystallized cognitive abilities, and other proxies of enriched earlier experiences such as educational quality. According to the CAR model, resilience is a dynamic developmental process relying on cognitive capacities developed in early life (Yao & Hsieh, 2019). This may be particularly true within adults experiencing homelessness who face extreme healthcare inequity, overexposure to trauma, and social stigma, all of which may require greater reliance on coping capacities developed in early life to interact effectively with their current environment in the context of acquired cognitive impairments (Luchenski et al., 2018; Mullins et al., 2021). Crystallized cognition is interconnected with the concept of cognitive reserve, or the disconnect between brain pathology/damage and behavioral presentations, and oral reading abilities have been used in past literature as a proxy for both constructs (Cattell, 1963; Stern et al., 2009, 2023). Cognitive reserve is an established resilience factor against the development and progression of dementias (Alvares Pereira et al., 2022; Stern, 2009), which is particularly relevant to adults experiencing homelessness who have an elevated risk of developing dementia (Beard et al., 2022; Dintica et al., 2023). Future work should further examine crystallized cognitive abilities to determine whether it represents a robust contributor to psychological resilience among adults experiencing homelessness who have executive functioning impairments and comorbid mental health disorders. Future work is needed to replicate study findings using a more comprehensive cognitive battery to further explore the relationships between resilience and domain-specific cognition, particularly within the cognitive domains not measured here that have been shown to relate to resilience such as verbal fluency and semantic retrieval (Deng et al., 2018; Laird et al., 2019; Simeon et al., 2007).

There are several important limitations to consider within the current study. Only individuals residing in a large Canadian urban centre were recruited for this study, so the results may not generalize to rural communities or countries beyond Canada. Despite this, the final sample included a wide age range and an approximately even gender division, which increases the generalizability of study findings. Although the present sample was screened to ensure sufficient English fluency for testing, the linguistic diversity of the current sample may have influenced the measurement of important study constructs. The subjective validity ratings made after the completion of the testing session are not as well-validated and may not be as rigorous as standard performance validity measures, which represents a limitation of the study. Similar approaches have been used in past research among individuals experiencing homelessness (e.g., Blumberg et al., 2024; Gicas et al., 2017, 2020) and sensitivity analyses indicated no difference in results with the inclusion of excluded participants; however, the use of an embedded validity indicator to ensure performance validity would have been preferable. Future work should aim to establish NIH Toolbox Cognitive Battery cutoff scores (e.g., Karr et al., 2024) that are appropriate for individuals with cognitive impairment and multimorbid physical and mental health concerns. Additionally, aside from the inclusion criteria (i.e., having experienced at least two episodes, at least seven consecutive nights, of homelessness or precarious housing in the past year), we did not collect information on the duration of homelessness experienced by each participant, which could help better characterize the study sample. Homelessness is typically a fluid experience, with individuals transitioning between different states of housing instability (Gaetz, 2012; Gaetz et al., 2014), which limits the analyses of current housing status to descriptive. Additional research suggests there are similar unmet needs and vulnerabilities across individuals experiencing homelessness and housing instability (Argintaru et al., 2013). Given the high rate of multimorbid mental illness in the study sample, a number of psychiatric factors may have influenced the relations between domain-specific cognition and resilience; future work should better control for these important potentially confounding factors given the heterogeneity of multimorbid mental health disorders commonly found among adults experiencing homelessness (Aldridge et al., 2018; Roncarati et al., 2018; Vila-Rodriguez et al., 2013). The cross-sectional study design precludes statements of causality or directionality between resilience and cognitive functioning. Finally, despite the strong psychometric properties of CD-RISC2, this measure is an abbreviated version of the full scale, the use of which may allow for a more comprehensive assessment of the construct of resilience (Connor & Davidson, 2003; Vaishnavi et al., 2007). Additionally, CD-RISC2 relies exclusively on self-reported resilience, so objective approaches to resilience that are independent of participants’ judgment and defined by external observation (e.g., resilience index based on objective measures of daily functioning) may yield differential results (Jones, 2019). Future research should include objective and subjective measures of resilience to expand our knowledge of how domain-specific cognition relates to psychological resilience in adults experiencing homelessness or precarious housing.

This study is the first to identify oral reading abilities as a novel correlate of psychological resilience within adults experiencing homelessness or precarious housing, which may reflect an association between greater enrichment in early life experiences and better coping skills in the face of adversity among adults experiencing homelessness. Better delineation of the cognitive correlates of resilience can help to identify modifiable targets for interventions that aim to improve quality of life and everyday outcomes in adults experiencing homelessness or precarious housing (Greenberg et al., 2019; Mejia-Lancheros et al., 2021). There is an urgent need to improve the everyday lived experience of this population given the elevated rate of multimorbid mental and physical illness and premature mortality associated with being unhoused (Aldridge et al., 2018). By enhancing both cognition and resilience, individuals experiencing homelessness may be better equipped to navigate challenging life situations and improve their overall mental health and well-being.

## Supplementary Material

Supplementary_Table_acaf018
