# Supplementary material for: Cognitive Correlates of Resilience in Adults Experiencing Homelessness
Source: Arch Clin Neuropsychol. 2025 Mar 4;40(6):1158–69. doi: 10.1093/arclin/acaf018 (PMC12378357; doi:10.1093/arclin/acaf018)
Supplement: Supplementary_Table_acaf018 [file supplementary_table_acaf018.docx]

**Supplementary Table 1**

*Full Model Regression Results with Resilience as the Outcome (N = 88)*

| Predictors | Estimate | *SE* | 95 % CI | | *p* |  |
| --- | --- | --- | --- | --- | --- | --- |
|  |  |  | *LL* | *UL* |  |  |
| *Model 1: Dimensional Change Card Sort* | | | | | |  |
| Female Gender | 0.25 | 0.40 | -0.54 | 1.03 | .537 |  |
| Age | < 0.01 | 0.01 | -0.02 | 0.03 | .788 |  |
| Education | 0.01 | 0.07 | -0.14 | 0.15 | .906 |  |
| Substance Misuse | 0.33 | 0.40 | -0.47 | 1.13 | .414 |  |
| **HADS** | **-0.13** | **0.03** | **-0.18** | **-0.07** | **< .001** |  |
| LSNS-6 | 0.01 | 0.03 | -0.05 | 0.07 | .739 |  |
| Dimensional Change | 0.06 | 0.14 | -0.21 | 0.34 | .642 |  |
| *Model 2: Flanker* | | | | | |  |
| Female Gender | 0.30 | 0.38 | -0.46 | 1.06 | .431 |  |
| Age | < 0.01 | 0.01 | -0.02 | 0.03 | .718 |  |
| Education | 0.01 | 0.07 | -0.13 | 0.16 | .844 |  |
| Substance Misuse | 0.35 | 0.39 | -0.44 | 1.13 | .381 |  |
| **HADS** | **-0.13** | **0.03** | **-0.18** | **-0.07** | **< .001** |  |
| LSNS-6 | 0.01 | 0.03 | -0.05 | 0.07 | .771 |  |
| Flanker | 0.13 | 0.18 | -0.24 | 0.49 | .494 |  |
| *Model 3: List Sorting* |  |  |  |  |  |  |
| Female Gender | 0.22 | 0.38 | -0.54 | 0.98 | .569 |  |
| Age | 0.01 | 0.01 | -0.02 | 0.03 | .559 |  |
| Education | 0.01 | 0.07 | -0.13 | 0.15 | .870 |  |
| Substance Misuse | 0.38 | 0.39 | -0.39 | 1.15 | .329 |  |
| **HADS** | **-0.14** | **0.03** | **-0.19** | **-0.08** | **< .001** |  |
| LSNS-6 | < 0.01 | 0.03 | -0.06 | 0.06 | .975 |  |
| List Sorting | 0.09 | 0.05 | -0.02 | 0.21 | .112 |  |
| *Model 4: Pattern Comparison* | |  |  |  |  |  |
| Female Gender | 0.29 | 0.38 | -0.46 | 1.04 | .447 |  |
| Age | < 0.01 | 0.01 | -0.02 | 0.03 | .749 |  |
| Education | 0.01 | 0.07 | -0.13 | 0.15 | .873 |  |
| Substance Misuse | 0.31 | 0.39 | -0.46 | 1.08 | .421 |  |
| **HADS** | **-0.12** | **0.03** | **-0.18** | **-0.07** | **< .001** |  |
| LSNS-6 | 0.01 | 0.03 | -0.05 | 0.07 | .728 |  |
| Pattern Comparison | 0.02 | 0.01 | < -0.01 | 0.04 | .094 |  |
| *Model 5: Picture Sequence* | |  |  |  |  |  |
| Female Gender | 0.29 | 0.38 | -0.48 | 1.05 | .454 |  |
| Age | 0.01 | 0.01 | -0.02 | 0.03 | .706 |  |
| Education | < 0.01 | 0.07 | -0.14 | 0.15 | .953 |  |
| Substance Misuse | 0.36 | 0.39 | -0.42 | 1.14 | .357 |  |
| **HADS** | **-0.13** | **0.03** | **-0.18** | **-0.07** | **< .001** |  |
| LSNS-6 | 0.01 | 0.03 | -0.05 | 0.07 | .763 |  |
| Picture Sequence | 0.14 | 0.21 | -0.28 | 0.55 | .514 |  |
| *Model 6: Picture Vocabulary* | |  |  |  |  |  |
| Female Gender | 0.09 | 0.39 | -0.68 | 0.86 | .815 |  |
| Age | -0.01 | 0.01 | -0.03 | 0.02 | .566 |  |
| Education | 0.01 | 0.07 | -0.13 | 0.15 | .927 |  |
| Substance Misuse | 0.15 | 0.40 | -0.64 | 0.94 | .710 |  |
| **HADS** | **-0.13** | **0.03** | **-0.18** | **-0.08** | **< .001** |  |
| LSNS-6 | 0.01 | 0.03 | -0.05 | 0.07 | .650 |  |
| **Picture Vocabulary** | **0.18** | **0.09** | **0.01** | **0.35** | **.041** |  |
| *Model 7: Oral Reading* | |  |  |  |  |  |
| Female Gender | 0.01 | 0.37 | -0.72 | 0.75 | .972 |  |
| Age | < -0.01 | 0.01 | -0.03 | 0.02 | .856 |  |
| Education | -0.05 | 0.07 | -0.18 | 0.09 | .502 |  |
| Substance Misuse | 0.39 | 0.37 | -0.34 | 1.12 | .294 |  |
| **HADS** | **-0.13** | **0.03** | **-0.19** | **-0.08** | **< .001** |  |
| LSNS-6 | 0.01 | 0.03 | -0.05 | 0.07 | .780 |  |
| **Oral Reading** | **0.18** | **0.05** | **0.07** | **0.29** | **.001** |  |
| *Model 8: All Cognitive Subtests* | |  |  |  |  |  |
| Female Gender | -0.05 | 0.40 | -0.86 | 0.75 | .897 |  |
| Age | -0.01 | 0.02 | -0.04 | 0.02 | .647 |  |
| Education | -0.04 | 0.07 | -0.19 | 0.10 | .551 |  |
| Substance Misuse | 0.28 | 0.41 | -0.54 | 1.09 | .501 |  |
| **HADS** | **-0.13** | **0.03** | **-0.19** | **-0.07** | **< .001** |  |
| LSNS-6 | 0.01 | 0.03 | -0.05 | 0.07 | .716 |  |
| Dimensional Change | < 0.01 | 0.15 | -0.30 | 0.30 | .978 |  |
| Flanker | -0.15 | 0.21 | -0.58 | 0.27 | .483 |  |
| List Sorting | 0.01 | 0.07 | -0.14 | 0.15 | .933 |  |
| Pattern Comparison | 0.02 | 0.01 | -0.01 | 0.04 | .189 |  |
| Picture Sequence | -0.03 | 0.21 | -0.45 | 0.39 | .888 |  |
| Picture Vocabulary | 0.07 | 0.10 | -0.14 | 0.28 | .511 |  |
| **Oral Reading** | **0.16** | **0.06** | **0.03** | **0.28** | **.018** |  |
| *Model 9: Fluid Cognitive Composite* | | |  |  |  |  |
| Female Gender | | 0.21 | 0.38 | -0.54 | 0.97 | .576 |
| Age | | 0.01 | 0.01 | -0.02 | 0.04 | .418 |
| Education | | 0.03 | 0.07 | -0.11 | 0.18 | .634 |
| Substance Misuse | | 0.32 | 0.39 | -0.46 | 1.09 | .416 |
| **HADS** | | **-0.13** | **0.03** | **-0.18** | **-0.08** | **< .001** |
| LSNS-6 | | < -0.01 | 0.03 | -0.07 | 0.06 | .939 |
| Fluid Cognition | | 0.02 | 0.01 | < -0.01 | 0.05 | .088 |
| *Model 10: Crystallized Cognitive Composite* | | | |  |  |  |
| Female Gender | | -0.03 | 0.38 | -0.78 | 0.72 | .936 |
| Age | | -0.01 | 0.01 | -0.03 | 0.02 | .479 |
| Education | | -0.02 | 0.07 | -0.16 | 0.12 | .774 |
| Substance Misuse | | 0.19 | 0.37 | -0.56 | 0.93 | .618 |
| **HADS** | | **-0.13** | **0.03** | **-0.19** | **-0.08** | **< .001** |
| LSNS-6 | | 0.01 | 0.03 | -0.05 | 0.07 | .705 |
| **Crystallized Cognition** | | **0.06** | **0.02** | **0.02** | **0.10** | **.002** |
| *Model 11: Crystallized–Fluid Difference Score* | | | |  |  |  |
| Female Gender | | 0.27 | 0.39 | -0.51 | 1.04 | .494 |
| Age | | < 0.01 | 0.02 | -0.03 | 0.03 | .956 |
| Education | | < 0.01 | 0.08 | -0.15 | 0.16 | .954 |
| Substance Misuse | | 0.39 | 0.39 | -0.39 | 1.18 | .323 |
| **HADS** | | **-0.13** | **0.03** | **-0.18** | **-0.07** | **< .001** |
| LSNS-6 | | 0.01 | 0.03 | -0.05 | 0.07 | .761 |
| Crystallized–Fluid | | < 0.01 | 0.01 | -0.02 | 0.03 | .748 |
